# Reduced volume SIB-IMRT/IGRT to head and neck cancer in elderly and frail patients: outcome and toxicity

**DOI:** 10.1186/s13014-016-0711-x

**Published:** 2016-10-06

**Authors:** Christoph Straube, Steffi U. Pigorsch, Hagen Scherb, Jan J. Wilkens, Henning Bier, Stephanie E. Combs

**Affiliations:** 1 Department Radiation Oncology, Klinikum rechts der Isar, Technical University of Munich (TUM), Ismaninger Str. 22, 81675 Munich, Germany; 2 Institute of Computational Biology, Helmholtz Zentrum München, Deutsches Forschungszentrum für Gesundheit und Umwelt (GmbH), Ingolstädter Landstr. 1, 85764 Neuherberg, Germany; 3 Institute of Innovative Radiotherapy (iRT), Helmholtz Zentrum München, Deutsches Forschungszentrum für Gesundheit und Umwelt (GmbH), Ingolstädter Landstr. 1, 85764 Neuherberg, Germany; 4 Department Otorhinolaryngology, Head and Neck Surgery, Technical University of Munich (TUM), Ismaninger Str. 22, 81675 Munich, Germany

## Abstract

**Background:**

Especially elderly and frail patients have a limited ability to compensate for side effects of a radical treatment of head and neck malignancies. Limiting the target volume to the macroscopic disease, without prophylactic nodal irradiation, might present a feasible approach for these patients. The present work therefore aims evaluating an IMRT/IGRT –SIB concept for safety and efficacy.

**Methods:**

The study retrospectively enrolled 27 patients with head and neck cancers treated between 01/2012 and 05/2015. We evaluated patient files for clinical status, concomitant diseases, treatment side, and treatment volumes as well as for side effects and tumor responses. To describe efficacy and risk factors for worse outcome and higher grade toxicities, we performed cox regression analysis as well as Kaplan-Meier survival time analysis.

**Results:**

Median survival was 181 days, 75 % patients showed an early local response at six weeks of follow up. Most patients developed mild to moderate acute toxicities, only one patient with grade IV mucositis was seen. The grade of toxicities was correlated to the size of the PTV. Concomitant diseases, metastatic disease, and G3 Grading were indicators for worse prognosis.

**Conclusion:**

The IMRT/IGRT SIB concept is a safe and feasible radiotherapy concept for patients not able or not willing to undergo radical treatment.

## Introduction

Around 48.000 new cases of oral cavity and pharyngeal cancer are diagnosed within the United States every year, causing about 9500 patients deaths annually [1]. For Germany the Robert-Koch-Institute counted 9.300 newly diagnosed cancers of the oral cavity and pharynx in men and 3.650 in women; for laryngeal cancer, 3.110 cases in men and 490 cases in women were expected for first diagnosis in 2012 [2]. Radical treatment, including multimodal approaches with surgery, radiotherapy (RT) and chemotherapy, or combinations of these, can offer significant advantage in local control as well as in overall survival compared with best supportive care. However, this beneficial results can be associated with side effects, which are related to the treated region, especially the large amount of mucosa included into the target volume [3–5].

There is some controversy regarding the subgroup of elderly and frail patients; depending on the tumor stage, surgery and/or RT might be indicated, however, based on the reduced overall prognosis, the real value of radical local or palliative systemic treatment remains unclear [6, 7]. Taking the short survival of few weeks up to 4 months of patients with stage IV disease under best supportive care into consideration, it can be discussed that a reduction of treatment time is beneficial. However, this must outweigh any increase in short or mid-term side effects, which can be associated with higher local doses related to shortened treatment times [8]. Consequently, a RT regimen for elderly and frail patients should have a lower burden of side effects, and, taking the shorter overall survival times into account, should need a shorter total treatment time [9]. Several palliative regimens, most of them using 2D and 3D-techniques, have been evaluated in prospective clinical trials [10–12]. However, use of modern techniques such as IMRT and IGRT might decrease side effects without decreasing the efficacy [13].

In the present manuscript we report on our experiences with a hypofractionated IMRT/IGRT SIB-regimen offered to patients in reduced overall performance status based on individual decision making in interdisciplinary discussion, with special focus on patient prognosis and treatment toxicity.

## Patients and methods

### Patients

Between 01/2012 and 05/2015, 450 patients were treated for head and neck malignancies at the Department for Radiation Oncology at the Technical University in Munich, Germany; all patients are included into our prospective patient database of the department. This prospective patient registry was searched for patients with head and neck malignancies treated in palliative intent between January 2012 and April 2015. The project (project number 113/15) was approved by the local ethics committee of the Medical Faculty at the Technical University of Munich.

Only patients with intensity modulated radiotherapy (IMRT or volumetric modulated arc therapy - VMAT) limited to gross disease who were treated with simultaneous integrated boost (SIB up to 54 Gy) were included. Tumour characteristics, patient history, performance status, clinical course, response, toxicities, and survival times were extracted from the database and the patient’s medical records.

Out of all patients with head and neck cancer, 27 patients fulfilled the above criteria and were treated with the hypofractionated regimen with a decreased volume RT up to a total dose of 40 Gy with SIB to 54 Gy.

Twenty-four patients were male (89 %), 3 patients were female (11 %). Median age at diagnosis was 69.2 years (49.3 to 87.8 years). All besides two patients had primary tumour sites of the head and neck, no patient with nasopharyngeal cancer was included. Two patients were treated for nodal diseases of cervical CUP-syndrome. Median Karnofsky Performance Status (KPS) before onset of radiotherapy was 70 % (40 % to 100 %). The majority of patients (21 of 27) were hospitalized for RT to ensure compliance and to guarantee for effective supportive care. Detailed patients’ characteristics are summarized in Table 1.

**Table 1:** Patient characteristics

Characteristics	All patients	
	Number	Percentage
Age at diagnosis, median (range) [years]	69.2 (49-87)	
Sex		
male	24	88.9
female	3	11.1
Karnofsky performance status		
100-90	3	11.1
80-70	17	63.0
60-50	6	22.2
40-30	1	3,7
BMI, median (range) [kg/m^2^]	22.7 (17.8-35.9)	
Comorbidities, median (range)	4 (1-15)	
Cardiac and Vascular-disease^b^	15	55.6
Lung-disease^c^	8	29.6
Liver-disease	4	14.8
Diabetes	2	7.4
Alcohol abuse	15	55.6
Dementia	5	18.5
Cachexia^d^	8	29.6
Charlson comorbidity Index		
2-4	13	48.1
5-8	10	37.0
9-12	4	14.8
Laboratory findings		
decreased Cholin Esterase^a^	11	
increased CRP (>1.0 g/dl)	12	44.4
UICC Stage		
IVa	14	51.8
IVb	4	14.8
IVc	9	33.3
Histology		
Squamous cell carcinoma	25	92.6
Adenocarcinoma	1	3.7
Mucoepidermoid carcinoma	1	3.7
Reason for non-radical treatment^b^		
Tumor size not suitable for radical treatment	5	18.5
Metastatic disease	9	33.3
Age and/or severe comorbidity	21	77.8
Re-Radiotherapy	1	3.7

^a^ 14 patients analysed

^b^ Including coronary heart disease, myocardial infarction, arrhythmia, congestive heart failure

^c^ Including emphysema, COPD, high frequency of pneumonia

^d^ Defined as progressive, unwanted weight-loss

Distressing symptoms at the onset of radiotherapy were pain (2 cases), bleeding (2 cases), feeding problems (6 cases), dyspnoea (7 cases), functional impairment due to large primary or nodal disease (11 cases) and ulceration (7 cases). Some patients were admitted with more than one distressing symptom. In 4 cases, no distressing symptoms were documented.

Decision for reduced volume radiotherapy was made in an interdisciplinary setting; arguments for this concept were large gross tumor volume precluding curative treatment in 4 patients and because of metastatic disease in 10 patients. 21 of 27 patients suffered from severe concomitant diseases. In one patient, decision for limited volume radiotherapy was made because of a former RT within the same region. Another patient was treated in non-curative intent at an age of 85 with severe frailty and reduced KPS (85 year old patient with supra-glottic laryngeal cancer).

### Radiotherapy

All patients were planned based on 3 mm sliced, contrast enhanced CT scans. A head and shoulder mask was used for fixation in all cases. If MRI was available for target volume definition (19 of 27 cases), the MRI was co-registered to the planning CT scans. Gross Tumor Volume (GTV) for primary tumor and macroscopic suspicious cervical lymph nodes was delineated, GTV + 10 mm was defined as clinical target volume (CTV1). The planning target volume (PTV) was added depending on the setup and overall repositioning accuracy and was between 3 and 5 mm. Dose prescription was 50 % of PTV receiving the prescribed dose of 40 Gy (D_50_ = 40 Gy) in 20 fractions. Simultaneous integrated boost (SIB) of 54 Gy in 20 fractions was applied to the GTV + 5 mm margin (CTV_SIB). No prophylactic nodal irradiation was performed. All patients were treated 5 times per week with 6 or 15 MeV photons on a linear accelerator (Varian, Switzerland). The total dose is calculated to biologic equivalent dose of EQD2 of 68 to 70 Gy using an α/β ratio of 10 and taking a reduction of total treatment time by 21 days, compared to a treatment time of 49 days in radical treatments (0.6 Gy per day) into account [14].

Target volumes were delineated using the iplan RT v4.1.1 Planning software (BrainLab, Feldkirchen Germany) in all cases VMAT-plans or IMRT-plans were calculated using Varian Aria External Beam Planning software Version 13.

For all patients treated by SIB techniques a daily cone beam CT imaging and online correction of positioning mismatches were used.

### Follow up

All patients were included into a strict follow-up regimen including clinical and imaging-based follow-up. Generally, a first follow-up visit is scheduled six weeks after completion of RT, thereafter in 3-months intervals or as needed clinically. Due to the palliative setting, follow-up was often based on a limited and clinically necessary follow-up, and often no visit to the hospital was possible. The median follow up time, defined as time between last fraction of RT and last contact to the patient, was short with 104 days [median; range 1 to 940 days]. Response criteria were based on imaging data that were collected in 18 cases 6 weeks after the end of radiotherapy. Additionally, one patient who died from sigma perforation underwent autopsy. In one additional case, response was assessed after 3 months. We preferred MRI (13 cases) imaging to CT imaging (6 cases) for early response assessment. Furthermore, clinical assessment was done. Classification of early response was made according to the RECIST criteria, defining a complete response (CR) as a disappearance of all target lesions, and a reduction in the short axis to <10 mm in any pathological lymph nodes. A partial response (PR) was defined as at least a 30 % decrease in the sum of diameters of target lesions, taking as reference the baseline sum diameters. Progressive Disease (PD) is scored with a minimum of 20 % increase in the sum of diameters of target lesions, and Stable Disease (SD) showing neither sufficient shrinkage to qualify for PR nor sufficient increase to qualify for PD [15].

### Outcome evaluation and statistical analysis

Overall survival was defined as time between indication for palliative radiotherapy and death. In case of unknown survival times we contacted the general practitioners to gain further information. If the patient was still alive at the last follow up, the survival times were censored to the date of the last contact. Statistical analysis was done using the SAS LIFETEST procedure (SAS version 9.3). Kaplan Meier estimates of survivor functions and log-rank tests comparing underlying hazards and median survival times of grouped right-censored data were calculated. The SAS procedure PHREG was employed to estimate the hazard ratios (HR), corresponding 95 %-confidence limits, and two-sided p-values of the observed survival time data. The backward selection feature of procedure PHREG was used to identify important prognostic factors among the candidate co-variables under study. For this, the impact of age, performance status, number of comorbidities, body mass index, and cancer related risk factors such as inflammation parameters like CRP and metastasized stage was considered.

Spearman correlation coefficients were calculated to correlate treated volumes with the observed grade of toxicities.

If percentages were reported, e.g. toxicities, the percentages were calculated to the number of patients that were in follow up at the time point of relevance and not to the total number of patients in the study. This was done in order to avoid underreporting of toxicities.

## Results

### Tolerability and side effects

Therapy was completed as scheduled in 24 of 27 (89 %) cases. In one case the regimen was changed towards single doses of 3 Gy because of rapid progressive disease. The RT of the remaining two patients was ended previously due to worsening of concomitant disease and reduction of KPS. However, overall an acceptable tolerability of the RT regimen can be seen.

Toxicities were scored base on the CTCAE version 4.03 criteria. Acute toxicities were seen on most patients, however no severe acute side effects > CTCAE Grade IV were observed, only 1 patient developed Grade IV CTCAE mucosal bleeding (Table 2). Dysphagia was the most common severe side effect with the need of feeding tubes or parenteral feeding in 11 of 27 cases (41 %) at the end of RT. Noteworthy, we recommend in general early feeding tube implantation before onset of radiotherapy. Only 7 of the 11 patients (26 % of 27 patients treated) newly developed high grade dysphagia during the course of RT, while 5 patients were dependent on their feeding tube already before treatment. In three cases, initial disease dependent dysphagia resolved already during treatment and oral feeding was started. Weight loss was moderate with a loss of 3.2 kg during the course of radiotherapy (average weight loss 4 % of initial body weight, ranging from 10 % gain to a loss of 19 %). Opioid use was necessary in 13 of 27 cases during radiotherapy. Of note, dysphagia, skin toxicity, and mucositis where significantly related to the volume of the treated PTV (Table 5).

**Table 2:** Acute toxicity, according to CTCAE v4.03

	All Patients	
	Number	Percentage
Dermatitis		
Grade 0	4	14.8
Grade 1	11	40.7
Grade 2	12	44.4
Mucositis		
Grade 0	3	11.1
Grade 1	4	14.8
Grade 2	11	40.7
Grade 3	8	29.6
Grade 4	1	3.7
Dysphagia		
Grade 0	3	11.1
Grade 1	5	18.5
Grade 2	8	29.6
Grade 3	11	40.7
Pain at the end of treatment		
no pain	7	25.9
NSAR-Analgetics	3	11.1
weak opioids	4	14.8
strong opioids	13	48.2
Infections during treatment		
Pneumonia	6	22.2
others	4	14.8

In 18 of 27 patients, the six week toxicity data (first follow-up) were available. No Grade III or IV mucositis or radiodermatitis was seen at this time point. Three patients reported about ongoing severe dysphagia with the need of feeding support. Opioid use was still necessary in 3 cases (Table 3).

**Table 3:** Late acute toxicity, 6-week after end of radiotherapy (according to CTCAE v4.03)

	18 Patients	
	Number	Percentage
Dermatitis at 6 weeks after RTx		
Grade 0	8	44.4
Grade 1	7	38.9
Grade 2	3	16.7
Mucositis at 6 weeks after RTx		
Grade 0	14	77.8
Grade 1	3	16.7
Grade 2	1	5.6
Dysphagia at 6 weeks after RTx		
Grade 0	8	44.4
Grade 1	4	22.2
Grade 2	3	16.7
Grade 3	3	16.7
Pain at 6 weeks after RTx		
no pain	15	83.3
NSAR-Analgetics	0	0
weak opioids	0	0
strong opioids	3	16.7

Long term follow up was available for 9 of 27 patients with no severe toxicity. Grade 3 dysphagia was present in one patient, however, this was not due to RT but to persistent gross disease interfering with swallowing function.

### Response and survival outcomes

**Fig. 1:**
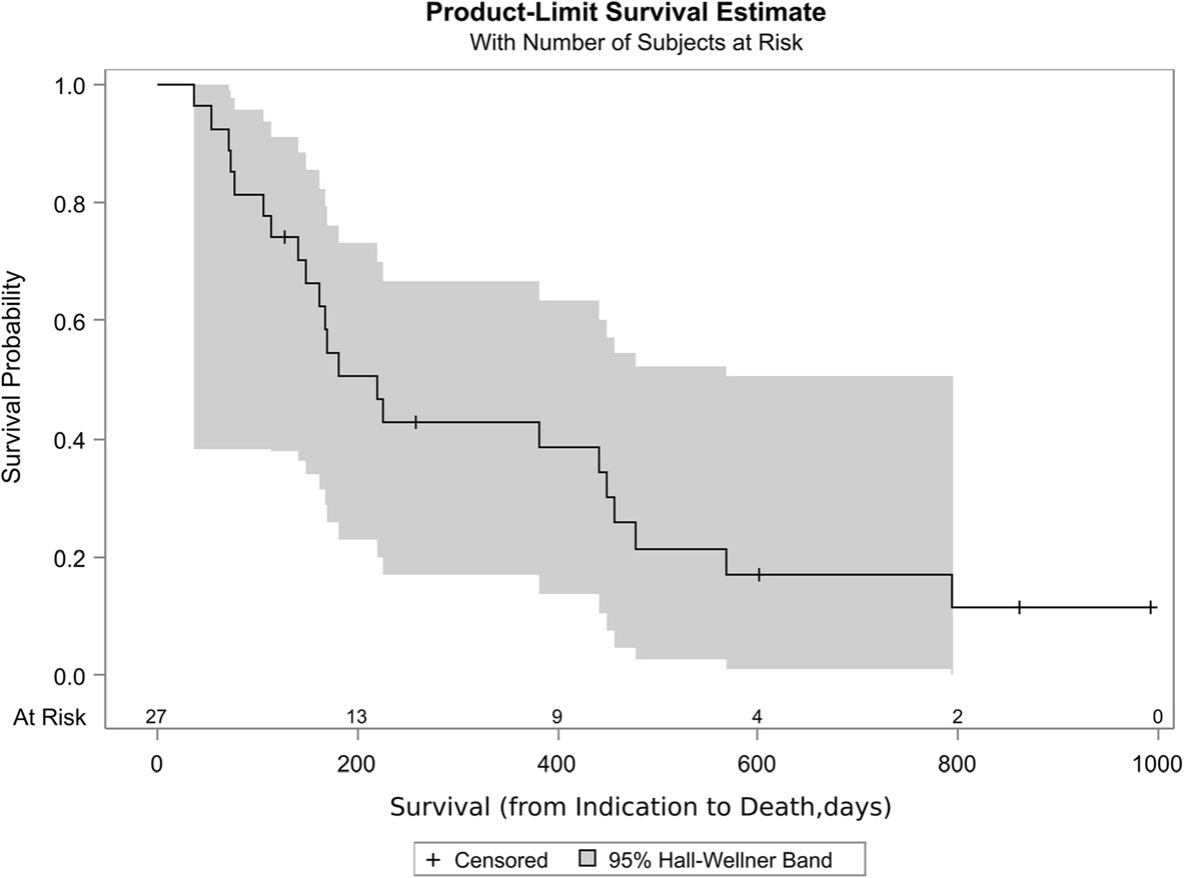
Kaplan-Meier Estimates of Survival for the Entire Population

Median survival was 181 days [range 55-999 days, 95 % CI: 92 to 269 days] from indication and 134 days [range 1-940 days, 95 % CI: 54 to 213 days] from end of RT (Fig. 1). The median duration of one course of RT was 28 days (range 10 to 34 days). Three of 27 patients (11 %), in whom the initially prescribed dose could not be reached, died within 60 days after the end of RT (6, 51 and 60 days). Seven patients (26 %) died before first scheduled follow up. There was no treatment related death. Nine Patients died due to progressive disease. In four cases (15 %), patient’s death was due to concomitant disease, in two of the cases sigma perforation was diagnosed by autopsy. Ten patients survived more than 300 days (range 341 to at least 940 days). At the time point of this analysis, 5 patients of the reported cohort are alive (80 to 940 days after the end of radiotherapy).

For 19 of 27 patients (70 %), the early response at 6 weeks after radiotherapy was available. Local response was achieved in 13 of these 19 patients (68 %). One patient presented with CR and 9 patients had a partial remission (PR) according to the RECIST 1.1 criteria. Two patients had stable disease (7 %), 2 further patient had local response but systemic progression (7 %), 4 patients presented with a RT-in-field-progression (15 %). One further patient died 6 weeks after radiotherapy due to a perforated sigma, autopsy showed a complete regression of all tumor masses.

### Co-factors and treatment outcome

**Fig. 2:**
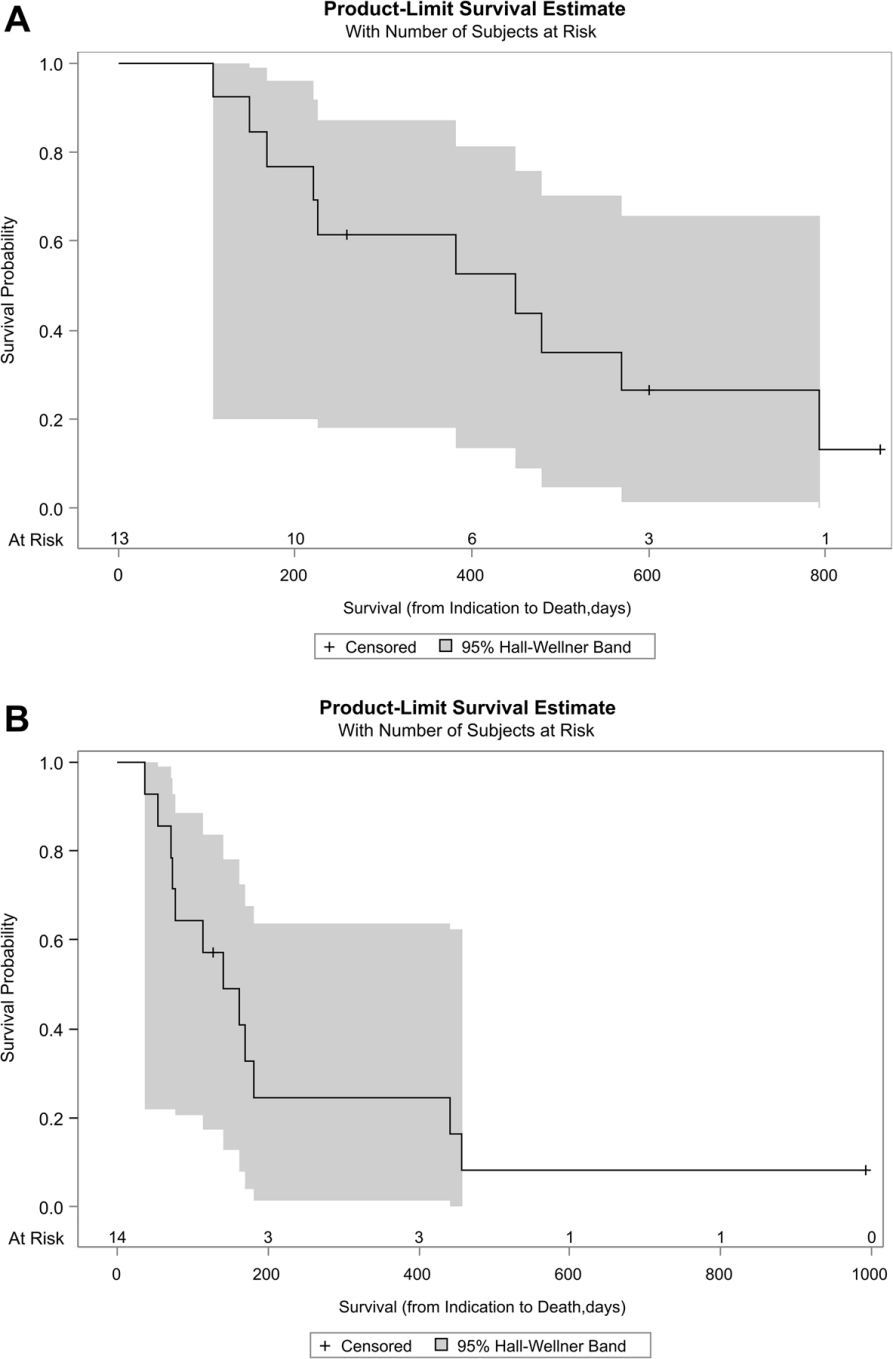
Kaplan-Meier Estimates of Survival in high-risk vs. low-risk Patients. Overall survival for patients with either ≤3 concomitant diseases or with >3 concomitant diseases and no tumor related risk factor (Panel **a**). Panel **b**: Overall survival for patients with > concomitant diseases and at least one tumor related risk factor

Cox regression analysis of short-term survival was significant (p < 0.05) for decreased BMI (<20 kg/m^2^ at time point of indication) and a decreased serum Cholin Esterase activity (CHE, lower than the age adjusted reference value). Neither a high Charlson Comorbidity Index (CCI), a decreased Karnofsky performance status (KPS <60 %) nor the number of concomitant diseases or the number of prescribed drugs influenced survival significantly. Patients with more than 3 concomitant diseases and the presence of at least one of four cancer related risk factors (metastatic disease, high-grade biology, biochemical markers for pre-cachexia, such as CRP <1 and/or lowered serum Cholin Esterase activity; high-risk) were compared to patients with either less than 3 concomitant diseases or more than 3 concomitant diseases but no cancer related risk factors (low-risk). Patients with a high-risk-profile had a significant shorter survival-time than patients with a low risk profile (median survival 141 vs. 450 days, CI 72-181 and 168-793 days, respectively; Fig. 2a and b; Table 4).

**Table 4:** Cox regression analysis of factors influencing survival

Variable	HR	95 %-LL	95 %-UL	*p*-value
Age >70	0.2560	0.061	1.079	0.0634
G3 vs G1 + 2	0.4380	0.148	1.296	0.1358
ChE reduced yes vs. No	0.2350	0.069	0.800	0.0205
BMI (<20 vs. >20 kg/m^2^)	53.120	1.265	22.311	0.0226
Low-Risk vs. High-Risk^a^	0.1540	0.044	0.541	0.0035
Charleston Comorb.-Index	0.9460	0.775	1.156	0.5886
Number of side diagnoses	11.670	0.976	1.396	0.0898
Number of medications	10.300	0.898	1.181	0.6758
CRP >1 vs <1	15.300	0.477	4.905	0.4745
M1 vs M0	24.990	0.901	6.928	0.0783
Karnofsky =60	21.770	0.522	9.077	0.2854

^a^ High-Risk-patient had more than 3 concomitant diseases and presented with at least one cancer related risk factor (biochemical sign for pre-cachexia by elevated CRP or decreased ChE-Level or decreased Albumin-Level; G3 grading; metastatic disease). Low risk patients had either more than 3 concomitant diseases but no such cancer related risk factor or less than 3 concomitant diseases but presence of cancer related factors

*ChE* Cholin Esterease serum activity

*CRP* C-Reactive Protein

## Discussion

For local control of head-and-neck tumors in elderly patients with significant comorbidities and overall reduced performance status, RT can be an effective treatment. To reduce overall treatment time as well as to reduce the amount of healthy tissue effected by the treatment, a shortened-course regimen with SIB was evaluated on 27 patients; overall, the treatment was very well tolerated without any significant severe short or mid-term side effects. However, target volume concept was associated with reduced margins compared to standard radiotherapy, with the main focus of local tumor control. Thus, diligent patient selection not to risk under-treatment is necessary.

With this aim, several groups have focussed on this special patient population. Especially with the advent of modern high-precision radiotherapy techniques, safe and normal-tissue sparing concepts with local dose escalation have become feasible. For patients with head and neck cancer who are deemed to be unsuitable for aggressive loco-regional treatment, several regimens for palliative radiotherapy, with and without concomitant chemotherapy, have been published so far [10, 12, 16–19]. Duration and intensity of RT-treatment regimens shows a huge variety, from 16 Gy in 16 fractions within 2 days to 60 Gy in 6 weeks [17, 20].

When deciding on such a regimen, which might be considered prospective palliation, exact staging, clinical workup and also clinical-oncological experience in patient assessment is required. Detailed patient history taking into account all other diseases, prior treatment or other important factors is essential. In most studies published previously, comorbidity was not reported. Moreover, it is a shortcoming of most guidelines for the treatment of head and neck cancer that there are no recommendations for treatment decision making taking into account comorbidities [21]. Nonetheless, concomitant diseases do have a strong effect on survival times, and cancer treatment itself might impact comorbidity outcome even when cancer specific survival is not affected [6, 22, 23].

A significant proportion of patients underwent limited volume radiotherapy for malignancies that did not yet cause distressing symptoms. In these patients, the treatment decision was made in order to avoid adverse symptoms that were deemed to result from further progressive local disease, termed “prospective palliation”. Temel et al. reported in 2010, that patients with end stage non-small-cell lung cancer had a better prognosis, when palliative treatment was started earlier [24]. In line with these results, patients undergoing aggressive early palliation in our cohort had a comparable better prognosis compared to patients that underwent treatment when distressing symptoms already developed (median survival 512 days vs. 121 days).

In our cohort, neither the CCI, nor age, nor KPS significantly affected treatment outcomes, possibly due to a high heterogeneity of comorbidities and relatively low number of cases. However, a decreased BMI and a decreased cholinesterase (ChE) activity were negative predictors for survival, markers that are related to cachexia and pre-cachexia [25]. It is known that tumor cachexia is a negative prognostic factor in various tumor types, predominantly head-and-neck or pancreatic cancer patients [26]. The analysis of a combination factor containing the number of comorbidities and the presence of cancer related risk factors was only descriptive, yet it significantly discriminated patients with very poor from patients with favourable outcome.

The palliative effect of a treatment is mainly related to the local response of the disease, and, vice versa, local tumor progression represents an important impact on the quality of life of patients with head and neck malignancies [13]. Tumor response at six weeks after radiotherapy could be assessed in 19 cases, with local response in 13 patients (68 %). As also UICC stage IVC patients were included, it is noteworthy to mention that two of these patients were in PD due to systemic or nodal disease while the irradiated tumors were in partial response, leading to a systemic response rate of 58 % (11/19 patients). Response rates have only been reported in patients with non-metastasized stages before onset of treatment. Hence, the local control-rate of 68 % should serve as a baseline to compare our results with others. Agarwal et al. reported about 73 % of patients in CR and PR after gross disease directed radiotherapy up to 40 Gy in 15 fractions. In difference to our study, also stage III patients were included into this cohort [19]. The same limitation should be considered when comparing the results from the “Hypo Trial”, where almost one third of patients where in stage I-III. The response rate was 80 %, however, the overall survival was 6.1 months, likely due to the high amount of patients with reasonable low performance status [10]. This is in line with the overall survival observed with 6 months observed in our study as well as with survival times after palliative intended radiotherapy reported in the literature, ranging between 5.7 and 7.2 months [11–13, 19].

The palliation of symptoms is negatively affected by the amount of toxicity caused by the treatment. In our cohort, confluent mucositis as one of the most distressing symptoms occurred in about 33 % of cases and lasted less than 6 weeks. The degree of mucositis was significantly related to the irradiated volume, with larger tumors being related to more severe side effects during treatment (Table 5). However, not only the degree of toxicity, which is usually confined to the highest degree observed, but also the area involved by toxicities should be considered. Avoiding prophylactic nodal irradiation was one first step to reduce this area of lower-grade toxicity. A second step was to reduce the safety margins by implementing new techniques such as IGRT and IMRT, in the regimen presented here to 10 mm safety margin. In almost all reports 2D or 3D planning without daily image guidance was used, leading to safety margins used mostly ranging from 1.5 to 2.0 cm [10, 11]. Van Beek et al. retrospectively compared 2D, 3D and IMRT irradiated palliative patients. Grade 3-4 mucositis occurred more often in patients treated with older techniques than patients treated with IMRT (44 % vs. 26 %), concluding that IMRT should be considered also for palliative treatments [13].

**Table 5:** Spearman correlation coefficients of treated volumes and acute toxicity

Spearman Correlation Coefficients				
		Mucositis	Dysphagia	Skin toxicity
PTV [cm^3^]	r	0.4398	0.4865	0.5261
	p	0.0217	0.0101	0.0048

This report has several limitations. Firstly, there is a significant heterogeneity within the study population, including patients with less symptoms but a high amount of concomitant diseases as well as patients with acute symptoms. Both groups had in common, due to the additive effects of oncologic and non-oncologic diseases, to be not able to undergo aggressive loco-regional treatment. The treatment decisions therefore were made on an individual basis. Of course, when clinically possible, conventionally accepted RT or RCHT is applied, and the presented short-course regimen is reserved for elderly and very comorbid patients. Moreover, no patient reported data of quality of life (QoL) is included into the present analysis. In the absence of patient reported quality of life data, the good tumor response as well as the acceptable toxicity profile can serve as surrogate parameters for the palliative efficacy of this regimen. Since patients are treated in several smaller centres or taken care of in palliative care centres or at home after treatment, it was difficult to assess all data in a standardized fashion since not all patients were present themselves for clinical follow-up. Long term tumor response and PFS could not be calculated, as most of the patients were in best supportive care after the end of RT and therefore no diagnostic attempts were undertaken in these cases. On the other hand, all patients were treated with modern IMRT/IGRT in a highly standardized fashion. In spite of these arguments, the present data can show that such a regimen is safe and effective. Moreover, the data show overall safety and very good tolerability. The low overall survival rates confirm the fact that death was not related to local progression and underline that such a regimen is justified in selected patients and should be kept in mind during RT planning and interdisciplinary decision making.

## Conclusion

The presented SIB-regimen offers an effective local treatment with manageable toxicity. By reducing the total treatment time to 4 weeks, and limiting the treatment to gross disease, it should be considered for patients that are not able or not willing to undergo radical treatment. For patients with significant concomitant disease and tumor related risk factors such as G3 Grading, pre-cachexia or metastatic disease, even shorter treatments or best supportive care only might be taken into consideration.

## Abbreviations

BMI: Body mass index
CCI: Charlson comorbidity index
CHE: Cholin Esterase
CR: Complete response
CRP: C-reactive protein
CT: Computed tomography
CTCAE: Common terminology criteria for adverse events
CTV: Clinical target volume
CUP: Cancer with unknown primary
GTV: Gross tumor volume
Gy: Gray
HR: Hazard ratio
IGRT: Image-guided radiation therapy
IMRT: Intensity-modulated radiation therapy
KPS: Karnofsky performance score
PD: Progressive disease
PR: Partial response
PTV: Planning target volume
QoL: Quality of life
RCHT: Radio-chemo-therapy
RT: Radiation therapy
SD: Stable disease
SIB: Simultaneous integrated boost
VMAT: Volumetric arc therapy.
